# Vegetable and Fruit Intake and Fracture-Related Hospitalisations: A Prospective Study of Older Women

**DOI:** 10.3390/nu9050511

**Published:** 2017-05-18

**Authors:** Lauren C. Blekkenhorst, Jonathan M. Hodgson, Joshua R. Lewis, Amanda Devine, Richard J. Woodman, Wai H. Lim, Germaine Wong, Kun Zhu, Catherine P. Bondonno, Natalie C. Ward, Richard L. Prince

**Affiliations:** 1 School of Medicine and Pharmacology, Royal Perth Hospital Unit, University of Western Australia, Perth, WA 6000, Australia; jonathan.hodgson@ecu.edu.au (J.M.H.); c.bondonno@ecu.edu.au (C.P.B.); natalie.ward@uwa.edu.au (N.C.W.); 2 School of Medical and Health Sciences, Edith Cowan University, Joondalup, WA 6027, Australia; a.devine@ecu.edu.au; 3 Centre for Kidney Research, Children’s Hospital at Westmead, Sydney, NSW 2145, Australia; joshua.lewis@sydney.edu.au (J.R.L.); germaine.wong@health.nsw.gov.au (G.W.); 4 School of Public Health, Sydney Medical School, University of Sydney, Sydney, NSW 2006, Australia; 5 Centre for Epidemiology and Biostatistics, School of Public Health, Flinders University of South Australia, Adelaide, SA 5042, Australia; richard.woodman@flinders.edu.au; 6 School of Medicine and Pharmacology, QEII Medical Centre Unit, University of Western Australia, Perth, WA 6009, Australia; wai.lim@health.wa.gov.au (W.H.L.); kun.zhu@uwa.edu.au (K.Z.); richard.prince@uwa.edu.au (R.L.P.); 7 Department of Endocrinology and Diabetes, and Department of Renal Medicine, Sir Charles Gairdner Hospital, Perth, WA 6009, Australia; 8 School of Biomedical Sciences & Curtin Health Innovation Research Institute, Curtin University, Perth, WA 6102, Australia

**Keywords:** vegetables, fruit, cruciferous, allium, fracture, bone, postmenopausal women

## Abstract

The importance of vegetable and fruit intakes for the prevention of fracture in older women is not well understood. Few studies have explored vegetable and fruit intakes separately, or the associations of specific types of vegetables and fruits with fracture hospitalisations. The objective of this study was to examine the associations of vegetable and fruit intakes, separately, and specific types of vegetables and fruits with fracture-related hospitalisations in a prospective cohort of women aged ≥70 years. Vegetable and fruit intakes were assessed at baseline (1998) in 1468 women using a food frequency questionnaire. The incidence of fracture-related hospitalisations over 14.5 years of follow-up was determined using the Hospital Morbidity Data Collection, linked via the Western Australian Data Linkage System. Fractures were identified in 415 (28.3%) women, of which 158 (10.8%) were hip fractures. Higher intakes of vegetables, but not fruits, were associated with lower fracture incidence. In multivariable-adjusted models for vegetable types, cruciferous and allium vegetables were inversely associated with all fractures, with a hazard ratio (HR) (95% confidence interval) of 0.72 (0.54, 0.95) and 0.66 (0.49, 0.88), respectively, for the highest vs. lowest quartiles. Increasing vegetable intake, with an emphasis on cruciferous and allium vegetables, may prevent fractures in older postmenopausal women.

## 1. Introduction

Dietary patterns rich in vegetables and fruits may provide benefits to bone health [1,2,3,4,5]. A number of studies have explored the relationships of defined dietary patterns with fracture risk. Although the results of these studies are inconsistent [1,4,5,6,7], they do suggest that certain components of these diets may contribute to lower fracture risk [8,9,10]. A major contribution may be the high intake of vegetables and fruits which are a key attribute of all healthy dietary patterns. This concept is supported by the results of prospective studies in a variety of populations finding that higher vegetable and fruit intakes are associated with lower risk of fracture [2,3,9,11]. However, there is little data on the effects of specific vegetable or fruit types on fracture outcomes. It is possible that some vegetables and fruits may be more protective than others due to specific nutrients and bioactive compounds such as phytochemicals.

In this study, we explored the associations of vegetable and fruit intakes, separately, with 14.5 years fracture-related hospitalisations in a prospective cohort of postmenopausal women aged ≥70 years. We then examined the associations of specific types of vegetables and fruits with fracture outcomes.

## 2. Materials and Methods

### 2.1. Study Population

The population included women in the Perth Longitudinal Study of Aging in Women (PLSAW). The women were originally recruited to a 5-year, double-blind, randomised controlled trial of daily calcium supplementation to prevent fracture, the Calcium Intake Fracture Outcome Study (CAIFOS). The women were included on the basis of an expected survival beyond 5 years and not receiving any medication (including hormone replacement therapy) known to affect bone metabolism. This trial has been previously described [12]. The women (*n* = 1500) were recruited from the Western Australian general population of women aged ≥70 years by mail using the electoral roll, which is a requirement of Australian citizenship. At the completion of the 5-year trial, women were invited to participate in two follow-up observational studies. Total follow-up was 14.5 years. A total of 1485 women completed a food frequency questionnaire at baseline in 1998. Participants (*n* = 17/1485, 1.1%) with implausible energy intakes (<2100 kJ (500 kcal) or >14,700 kJ (3500 kcal)) were not included in the analysis. The current study then included 1468 women. All participants provided written informed consent. Ethics approval was granted by the Human Ethics Committee of the University of Western Australia. Both studies were retrospectively registered on the Australian New Zealand Clinical Trials Registry (CAIFOS trial registration number #ACTRN12615000750583 and PLSAW trial registration number #ACTRN12617000640303), and complied with the Declaration of Helsinki. Human ethics approval for the use of linked data was provided by the Human Research Ethics Committee of the Western Australian Department of Health (project number #2009/24). 

### 2.2. Dietary Assessment

Dietary intake was assessed at baseline (1998), 5 years (2003), and 7 years (2005) using a self-administered, semiquantitative food frequency questionnaire developed and validated by the Cancer Council of Victoria [13,14,15]. The women were supported when completing the questionnaire by a research assistant. Food models and food charts as well as measuring cups and measuring spoons were provided to ensure the accuracy of reported food consumption. The Cancer Council of Victoria calculated energy (kJ/day) and nutrient intakes by using the NUTTAB95 food composition database [16] and other sources where necessary. Intakes of individual food items were calculated in g/day. This included 24 vegetables and 11 fruits. The diet assessment analysis also provided estimates of protein, calcium, and alcohol intakes.

#### 2.2.1. Vegetable and Fruit Intake

Vegetable and fruit intake were calculated in serves per day. This was based on the 2013 Australian Dietary Guidelines of 1 serve of vegetables equivalent to 75 g and 1 serve of fruit equivalent to 150 g [17]. Serves per day were calculated as continuous variables and then categorised as discrete variables (vegetables: <2 serves, 2 to <3 serves, ≥3 serves; fruit: <1 serve, 1 to <2 serves, ≥2 serves). Estimations of vegetable intake did not include ‘Potatoes, roasted or fried, including hot chips’ as hot chips are not recommended as part of a healthy diet [17]. ‘Potatoes cooked without fat’ were included. Estimations of fruit intake did not include ‘Tinned or frozen fruit (any kind)’ or ‘Fruit juice’ as foods and drinks containing added sugars are not recommended as part of a healthy diet [17]. 

#### 2.2.2. Vegetable and Fruit Type

Vegetables were grouped into five types. These vegetable types were based on the 2013 Australian Dietary Guidelines [17] and modified slightly to include cruciferous vegetables (cabbage, brussel sprouts, cauliflower, and broccoli); allium vegetables (onion, leek, and garlic); yellow/orange/red vegetables (tomato, capsicum, beetroot, carrot, and pumpkin); leafy green vegetables (lettuce and other salad greens, celery, silverbeet, and spinach); and legumes (peas, greens beans, bean sprouts and alfalfa sprouts, baked beans, soy beans, soy bean curd, and tofu, and other beans). For fruit, classification of type included: apples and pears (pome fruit); oranges and other citrus (citrus); bananas; and other fruits (melon, pineapple, strawberries, apricots, peaches, mango and avocado). Intakes of vegetable and fruit types were calculated in g/day as continuous variables. Intakes of vegetable types were also categorised as discrete variables into quartiles of intake.

#### 2.2.3. Nutrient-Rich Foods Index

Overall diet quality was assessed using the Nutrient-Rich Foods Index by calculating nutrient density scores [18]. This index was adapted using the Nutrient Reference Values (NRVs) for Australia and New Zealand based on adult females aged >70 years [19]. The calculation of the Nutrient-Rich Foods Index in this cohort of older women has been described previously [20].

### 2.3. Fracture Outcome Assessment

Fracture-related hospitalisations were retrieved from linked data via the Western Australian Data Linkage System (Department of Health Western Australia, East Perth, Australia) for each participant from their baseline visit until 14.5 years after their baseline visit. Fracture-related hospitalisations were identified from the Hospital Morbidity Data Collection which provides a complete record of participants’ primary diagnosis at hospital discharge using coded data from all hospitals in Western Australia. Fracture-related hospitalisations were defined using the International Statistical Classification of Diseases and Related Health Problems, 10th Revision [21]. Codes used for identification included S02-S92, M80, T02, T08, T10, T12, and T14.2. Fractures of the face (S02.2–S02.6), fingers (S62.5–S62.7), and toes (S92.4–S92.5), and fractures caused by motor vehicle injuries were excluded (external cause of injury codes V00–V99).

### 2.4. Baseline Characteristic Assessment

Questionnaires completed at baseline were used to assess values for potential confounding variables including age, physical activity, and smoking history. Participants were asked about participation in sport, recreation, and/or regular physical activities undertaken in the three months prior to their baseline visit [22,23]. The level of activity, expressed in kilojoules per day, was then calculated using a validated method applying the type of activity, time engaged in the activity, and the participant’s body weight [24,25]. Smoking history was coded as non-smoker or ex-smoker/current smoker if they had consumed >1 cigarette per day for more than 3 months at any time in their life. Body weight was measured using digital scales to the nearest 0.1 kg and height was assessed using a wall-mounted stadiometer to the nearest 0.1 cm, both whilst participants were wearing light clothes and without socks and shoes. Body mass index (BMI) (kg/m^2^) was then calculated. Treatment (placebo or calcium) over the 5 years of the CAIFOS trial was included as a covariate. Current medication use at baseline was used to assess prevalent diabetes mellitus. Medications were verified by participants’ general practitioner where possible and were coded (T89001–T90009) using the International Classification of Primary Care-Plus (ICPC-Plus) method which allows aggregation of different terms for similar pathologic entities as defined by the ICD-10 coding system [26]. Socioeconomic status (SES) was calculated using the Socioeconomic Indexes for Areas developed by the Australian Bureau of Statistics which ranked residential postcodes according to relative socio-economic advantage and disadvantage [27]. Participants were then coded into six groups from the top 10% most highly disadvantaged to the top 10% least disadvantaged [27]. Prevalent fractures were determined by self-reported fractures that had occurred after the age of 50 and prior to the participants’ baseline visit. Self-reported fractures were defined as a fracture due to a minimal trauma fall from less than 1 m that did not include fractures of the face, skull, and phalanges.

### 2.5. Statistical Analysis

Statistical analysis was performed using IBM SPSS Statistics for Windows, version 21.0 (IBM Corp., Armonk, NY, USA) and SAS software, version 9.4 (SAS Institute Inc., Cary, NC, USA). Statistical significance was set at a 2-sided Type 1 error rate of *p* < 0.05 for all tests. Descriptive statistics of normally distributed continuous variables were expressed as mean ± standard deviation (SD). Non-normally distributed continuous variables (physical activity, alcohol intake, allium vegetable intake, and all fruit types) were expressed as median and interquartile range. Categorical variables were expressed as number and proportion (%). Baseline characteristics were tested for differences across categories using one-way analysis of variance (ANOVA) for normally distributed continuous variables, the Kruskal-Wallis test for non-normally distributed variables, and the Chi-squared test for categorical variables.

The primary outcome of the study was first hospitalisation for fracture, with further analysis of hip fracture which accounted for more than one-third of all events. Complete follow-up was available for all participants that remained in Western Australia, which was likely to be almost all participants given their age. The follow-up time period for each participant commenced from their baseline visit date until the first fracture-related hospitalisation or loss to follow-up due to death or 174 months of complete follow-up. Cox proportional hazards modelling was used to assess the associations of vegetable and fruit intake variables with fracture outcomes. Two models of adjustment were used: age-adjusted and multivariable-adjusted. The multivariable-adjusted models included age, BMI, treatment code, prevalent diabetes mellitus, SES, physical activity, smoking history, and intakes of energy, protein, calcium, and alcohol. Associations were explored using continuous variables and then as discrete variables. We also tested for evidence of linear trends across categories of discrete variables using the median value for each category as continuous variables in separate Cox proportional hazards models. Cox proportional hazards assumptions were tested using log-log plots, which were shown to be parallel indicating that proportional hazards assumptions were not violated. For the primary analysis, we treated deaths as censored. This cause-specific approach meant that the hazard ratios could be interpreted as the risk of fracture for any time during follow-up assuming that the participant stayed alive for that duration of time. The calculated risk of fracture, therefore, assumed that the risk of fracture would have remained the same during the remainder of the follow-up period in those that died. This model was chosen as we have previously demonstrated that fruit intakes are associated with mortality risk in this cohort [28]. Based on the given data with 16,458 years of follow up and fracture event rates of 28.3% and 10.8%, respectively, for “all fractures” and “hip fractures”, the study had 80% power to detect a hazard ratio of 0.79 for all fractures and a hazard ratio of 0.69 for hip fractures, assuming a two-tailed type 1 error rate of alpha = 0.05.

#### Sensitivity Analyses

The relationships between cruciferous, allium, and total vegetables were investigated using Spearman Rank Order Correlation (rho). To examine whether the independent associations of cruciferous and allium vegetables were not due to collinearity with each other, a forward stepwise Cox proportional hazards model with all multivariable-adjusted variables as well as cruciferous (per 10 g/day) and allium (per 5 g/day) vegetables was analysed for all fractures. To examine whether the associations of cruciferous and allium vegetables were independent of total vegetable intake (per serve, 75 g/day), a forward stepwise Cox proportional hazards model with all multivariable-adjusted variables as well as intakes of cruciferous (per 10 g/day) and allium (per 5 g/day) vegetables, and total vegetable intake (per serve) was tested for all fractures. Intakes of cruciferous, allium, and total vegetables at baseline (1998), 5 years (2003), and 7 years (2005) were tested for differences using one-way repeated measures ANOVA. To account for change in cruciferous, allium, and total vegetable intakes, the average of the three time points (baseline, 5 years, and 7 years) for each vegetable variable were used in the multivariable-adjusted Cox proportional hazards model for all fractures. Since vegetable intake may be considered a surrogate marker of a healthier diet, we adjusted for diet quality using the Nutrient-Rich Foods Index in the multivariable-adjusted Cox proportional hazards model for all fractures. This was separately completed for cruciferous, allium, and total vegetable intakes. Lastly, since previous fractures can increase the risk of subsequent fracture [29], an interaction term between total vegetable intake and prevalent fracture was tested in the multivariable-adjusted Cox proportional hazards model. This was completed to evaluate if prevalent fracture had an impact on the relationship between total vegetable intake and all fractures. This test was repeated for intakes of cruciferous vegetables as well as intakes of allium vegetables. Lastly, as women may have commenced calcium or calcium plus vitamin D supplementation after the completion of the CAIFOS, we assessed whether the proportion of women who re-enrolled in the 5-year extension study (2003–2008) and commenced these supplements were different across vegetable serve categories. Furthermore, we analysed differences amongst women who commenced taking bisphosphonates from 1998–2003. It should be noted that data on calcium, calcium plus vitamin D, and bisphosphonate use were only available for *n* = 1007/1456 (69%) participants.

## 3. Results

### 3.1. Baseline Characteristics

Baseline characteristics of participants are presented according to all participants and categories of vegetable (Table 1) and fruit (Table 2) intakes. The mean age of participants at baseline was 75.2 years (SD 2.7 years). Significant differences were observed across vegetable intake categories for energy, protein, and calcium intakes (*p* < 0.001) (Table 1). Significant differences were observed across fruit intake categories for energy, protein, calcium, and alcohol intakes, physical activity, and smoking status (*p* < 0.01) (Table 2).

Mean (SD) intake for vegetables was 196.4 (79.1) g/day and 2.6 (1.0) serves per day, and for fruit it was 245.1 (128.6) g/day and 1.6 (0.9) serves per day. Mean (SD) intake (from highest to lowest) of vegetable types were: yellow/orange/red vegetables 51.8 (27.5) g/day; cruciferous vegetables 32.1 (21.9) g/day; legume vegetables 27.5 (18.7) g/day; and leafy green vegetables 18.6 (12.0) g/day. Median (interquartile range (IQR)) intake for allium vegetables was 6.2 (2.9–10.7) g/day. Median (IQR) intake (from highest to lowest) for fruit types were: apple and pears 54.4 (22.2–103.6) g/day; other fruits 49.5 (22.0–95.4) g/day; bananas 44.5 (18.5–72.6) g/day; and orange and other citrus fruits 35.5 (5.6–85.9) g/day. 

### 3.2. Fracture-Related Hospitalisation

Over 14.5 years (16,458 person-years) of follow-up, 415/1468 (28.3%) participants were hospitalised with a fracture, of which 158/1468 (10.8%) were hip fractures.

#### 3.2.1. Vegetable Intake 

Vegetable intake (per serve) was inversely associated with all fractures and hip fractures in age-adjusted and multivariable-adjusted models (*p* < 0.05) (Table 3). Compared with low intakes of vegetables (<2 serves/day), intakes of ≥3 serves/day were associated with a 27% lower hazard for all fractures (multivariable-adjusted *p*_trend_ = 0.023) and a 39% lower hazard for hip fractures (multivariable-adjusted *p*_trend_ = 0.037) (Table 3).

The associations between intakes of vegetable types and fracture-related hospitalisations in multivariable-adjusted models are presented in Table 4. Intakes of cruciferous and allium vegetables were inversely associated with all fractures (*p* < 0.05) (Table 4). The highest quartiles of cruciferous (multivariable-adjusted *p*_trend_ = 0.030) and allium (multivariable-adjusted *p*_trend_ = 0.003) vegetables in comparison to the lowest quartiles were associated with a 28% and 34%, respectively, lower hazard for all fractures. After additional adjustment for total vegetable intake, the hazard for all fractures was reduced and became non-significant for the highest quartile of cruciferous vegetables (multivariable-adjusted hazard ratio (HR): 0.80, 95% confidence interval (CI) 0.58, 1.10, *p*_trend_ = 0.160) compared to the lowest quartile. Allium vegetables, however, remained statistically significant (multivariable-adjusted HR: 0.70, 95% CI 0.52, 0.95, *p*_trend_ = 0.019). 

For hip fractures, the association of allium vegetables was borderline significant (*p* = 0.050), but did not reach significance for cruciferous vegetables (*p* = 0.157). Intakes of yellow/orange/red, leafy green, and legumes were not associated with all fractures (*p* > 0.05 for all) or hip fractures (*p* > 0.05 for all) (Table 4).

#### 3.2.2. Fruit Intake

Fruit intake (per serve) was not associated with all fractures (*p* > 0.05) and hip fractures (*p* > 0.05) (Table 5). The associations between intakes of fruit types and fracture-related hospitalisations in multivariable-adjusted models are presented in Table 6. All fruit types were not associated with all fractures (*p* > 0.05 for all) and hip fractures (*p* > 0.05 for all).

### 3.3. Sensitivity Analyses

There was a weak positive correlation between intakes of cruciferous and allium vegetables (Spearman’s rho = 0.11, *p* < 0.001) and a moderate positive correlation between intakes of allium and total vegetables (Spearman’s rho = 0.43, *p* < 0.001) and between intakes of cruciferous and total vegetables (Spearman’s rho = 0.52, *p* < 0.001). In a forward stepwise Cox proportional hazards model, which included all multivariable-adjusted variables and both cruciferous and allium vegetable intakes separately, age (per year increase, HR: 1.10, 95% CI: 1.06, 1.14, *p* < 0.001), BMI (per kg/m^2^ increase, HR: 0.98, 95% CI: 0.95, 1.00, *p* = 0.034), cruciferous vegetables (per 20 g/day increase, HR: 0.89, 95% CI: 0.81, 0.98, *p* = 0.019), and allium vegetables (per 10 g/day increase, HR: 0.82, 95% CI: 0.70, 0.97, *p* = 0.018) were associated with all fractures. In a forward stepwise Cox proportional hazards model, which included all multivariable-adjusted variables and total vegetable intakes as well as intakes of cruciferous and allium vegetables, age (per year increase, HR: 1.10, 95% CI: 1.06, 1.14, *p* < 0.001), BMI (per kg/m^2^ increase, HR: 0.98, 95% CI: 0.95, 1.00, *p* = 0.036), and total vegetable intake (per 75 g/day increase, HR: 0.87, 95% CI: 0.79, 0.96, *p* = 0.007) were associated with all fractures. 

Intakes of cruciferous, allium, and total vegetables were compared at baseline (1998), 5 years (2003), and 7 years (2005) in 986 participants. The mean (SD) at each time point for intakes of cruciferous, allium, and total vegetables are presented in Table S1. One-way repeated measures ANOVA were conducted for intakes of cruciferous, allium, and total vegetables and results confirmed a significant effect for time (Wilks’ Lambda *p* < 0.001 for all). Intake of cruciferous vegetables was 2.5 g/day (7.8%) lower at 7 years compared to intake at baseline. Intake of allium vegetables was 1.5 g/day (18.5%) lower at 5 years and 2.2 g/day (27.2%) lower at 7 years compared to intake at baseline. Total vegetable intake was 22.3 g/day (11%) lower at 5 years and 31.5 g/day (16%) lower at 7 years compared to intake at baseline. To account for this change, the average across baseline, 5 years, and 7 years was calculated individually for cruciferous, allium, and total vegetable intakes. The average values for cruciferous, allium, and total vegetable intakes were then entered separately into multivariable-adjusted Cox proportional hazards models for all fractures. This did not substantively alter the hazard ratios observed for baseline values and fracture-related hospitalisations (Table S2).

Adjustment for the Nutrient-Rich Foods Index in multivariable-adjusted models attenuated the associations of all fractures with intakes of total vegetables and cruciferous vegetables (*p* > 0.05 for both), but not allium vegetables (*p* = 0.027). No effect modification by prevalent fracture was observed for intakes of total vegetables (*p*_interaction_ = 0.617), cruciferous vegetables (*p*_interaction_ = 0.989), or allium vegetables (*p*_interaction_ = 0.482). Lastly, there were no differences in calcium (*n* = 12), calcium plus vitamin D (*n* = 393), or bisphosphonate (*n* = 80) use across vegetables serve categories amongst the *n* = 1007 women with available data (*p* > 0.05 for all).

## 4. Discussion

In this prospective cohort study of older women, we identified vegetable intakes, but not fruit intakes, to be associated with a lower hazard of all fractures and hip fractures. We also identified cruciferous and allium vegetable intakes to be individually associated with a lower hazard of all fractures, but not hip fractures. This may be due to the relatively low prevalence of hip fractures in this study and, therefore, the insufficient power to detect an association. The study was only powered to detect relatively large reductions in risk between food intake categories of around 20% for all fractures and 30% for hip fractures. The associations were independent of dietary and lifestyle factors known to be related to fracture risk.

Habitual intakes of vegetables and fruits, combined, have been associated with lower risk of fracture outcomes [2,5,6,30]. In particular, vegetable and fruit intakes have also been individually associated with lower risk of fracture outcomes [2,5,30]. A meta-analysis has identified vegetables, but not fruits, to be associated with reduced risk of hip fracture [31]. Our study is consistent with this meta-analysis, having demonstrated vegetable intake, but not fruit intake, to be associated with lower risk of fracture outcomes.

There are two main explanations why higher vegetable intakes could contribute to a lower risk of fracture. These include effects on bone mineral density and risk of falling. A number of studies have explored the link between vegetable and fruit intake and bone mineral density with inconsistent results. Some observational studies have found associations of vegetable and fruit intakes with bone mineral density [2,3,5,8,30,32,33], whilst others have not observed an association [34]. In addition, results of randomised controlled trials have not found consistent effects on biomarkers of bone turnover [35,36,37]. Evidence on the relationship of vegetable and fruit intakes with risk of falling are scant. Therefore, although there is strong evidence supporting beneficial effects of vegetables on fracture risk, the pathways and mechanisms responsible remain unclear.

To our knowledge, this is one of the first studies to investigate the associations of different types of vegetables and fruits with fracture outcomes in a population of older postmenopausal women. We demonstrated both cruciferous and allium vegetable intakes to be inversely associated with fracture risk, both of which were independent of each other. Cruciferous and allium vegetables contain an abundance of specific nutrients and phytochemicals that may benefit bone biology and subsequent fracture outcomes. For example, intakes of vitamin K (rich in cruciferous vegetables) have been shown to be inversely associated with hip fractures [38]. However, other studies have shown conflicting results [39]. Intakes of allium vegetables, in particular onions, have been shown to be associated with increased bone density in perimenopausal and postmenopausal women [40].

One mechanism through which phytochemicals may benefit bone biology is by the reductions in oxidative stress. Oxidative stress has been demonstrated to inhibit in vitro osteoblastic differentiation [41], and in human studies, relationships between excessive reactive oxygen species and bone loss have been observed [42,43]. Particular phytochemicals of interest found in both cruciferous and allium vegetables are organosulfur compounds. Sulforaphane, an organosulfur compound found abundantly in cruciferous vegetables, has been shown to inhibit in vitro human osteoclast differentiation [44], possibly due to the activation of nuclear factor-erythroid 2-related factor 2 (Nrf2) [45]. Nrf2 is a redox-sensitive transcription factor that regulates the expression of antioxidant proteins protecting against oxidative stress. In addition, sulforaphane has been shown to epigenetically stimulate osteoblast activity and reduce osteoclast bone resorption [46]. Park et al. [47] have also shown the alliin-containing vegetable, allium hookeri, to have in vitro and in vivo anabolic effects on bone formation. Allium hookeri is a widely consumed allium vegetable in Southeast Asia and is a rich source of alliin, an organosulfur compound that is also found abundantly in other allium vegetables such as garlic.

Strengths to this current study include the prospective design and population-based setting with ascertainment of verified fracture-related hospitalisations with almost no loss to follow-up. Participants of this study were representative of older women of the Australian population. The average vegetable serves of the women in this study were 2.6 serves which is the same for older Australian women aged ≥75 years [48]. In addition, there was also relatively detailed information on a number of known confounders including alcohol intake and socioeconomic status. Dietary information was collected at different time points and was collected using a validated and reproducible method of assessment. Limitations, however, need to be acknowledged. Participants of this study may have commenced taking calcium supplements or medications known to affect bone metabolism after the completion of the CAIFOS. However, we have demonstrated the proportion of participants that commenced taking calcium or calcium plus vitamin D supplements at the completion of the CAIFOS were similar across vegetable serve categories. In addition, medications known to affect bone metabolism are most likely prescribed after an osteoporotic fracture. Therefore, it is unlikely this would have influenced the interpretation of our findings. In addition, the dietary information, including habitual intakes of vegetables and fruits, were self-reported which may lead to misclassification of these variables. In addition, even though we adjusted for potential confounders such as dietary and lifestyle factors known to be associated with fracture risk, higher vegetable intakes may be a marker of a healthier lifestyle not completely captured by the lifestyle variables that we included as potential confounders in the multivariable-adjusted analyses. For example, participants consuming ≥3 serves/day of vegetable intake versus participants consuming <2 serves/day reported a 30% higher energy intake despite similar BMI. This suggests that they were more physically active. Even though the relationships of cruciferous, allium, and total vegetable intakes with fracture-related hospitalisations persisted after adjustment for energy intake and physical activity, the reported physical activity is relatively imprecise in comparison with these lifestyle factors. Although physical activity has been associated with geometric indices of bone strength in this cohort [49], reported physical activity using questionnaires are somewhat unreliable with the likelihood of under adjustment. We attempted to further address the possibility of higher vegetable intakes being a marker of a healthier lifestyle by adjusting for diet quality. This did attenuate the relationship for total and cruciferous vegetables, but not allium vegetables. The attenuation of the relationship for total and cruciferous vegetables and fracture-related hospitalisations indicates other constituents of a healthy diet at least partially explain the observed associations. It should also be noted that moderate correlations did exist between cruciferous and allium vegetables and total vegetable intakes. In addition, the inverse association between intakes of cruciferous vegetables and fracture-related hospitalisations was attenuated when adjusting for total vegetable intake. It is, therefore, possible that some of the effects seen for cruciferous vegetables may be due to their contribution to the overall increase in vegetable intake. Lastly, the observational nature of this study cannot establish a causal relationship, and the results of this study cannot be applied to younger cohorts and cohorts of older men.

## 5. Conclusions

The findings of this prospective cohort study indicate that habitual intakes of vegetables, but not fruits, are associated with a lower hazard of hospitalisations relating to fracture. These results are consistent with a recent meta-analysis of earlier studies [31]. We also found that intakes of cruciferous and allium vegetables were independently associated with lower hazard of all fractures, but not hip fractures. Increasing vegetable intake with a focus on consuming cruciferous and allium vegetables may reduce the risk of fracture in older postmenopausal women.

## Figures and Tables

**Table 1:** Baseline characteristics according to all participants and vegetable intake categories ^1^.

	All Participants	Vegetable Serve Intake ^2^			
		<2 Serves/Day	2 to <3 Serves/Day	≥3 Serves/Day	*p* Value ^3^
Number	1468	424	584	460	
Age, years	75.2 ± 2.7	75.2 ± 2.7	75.2 ± 2.7	75.0 ± 2.7	0.455
Body mass index (BMI) ^4^, kg/m^2^	27.2 ± 4.7	27.0 ± 4.8	27.2 ± 4.6	27.5 ± 5.0	0.233
Treatment (calcium) ^5^	755 (51.4)	202 (47.6)	309 (53.0)	244 (53.0)	0.175
Prevalent fracture ^5^	397 (27.0)	112 (26.4)	156 (26.8)	129 (28.0)	0.843
Prevalent diabetes mellitus	90 (6.1)	28 (6.6)	34 (5.8)	28 (6.1)	0.877
Physical activity ^4^, kJ/day	467.1 (0.0–855.4)	455.0 (0.0–886.3)	443.5 (159.4–836.9)	491.4 (157.7–852.0)	0.743
Smoked ever ^6^	546 (37.2)	156 (36.9)	224 (38.7)	166 (36.2)	0.698
Socioeconomic status ^7^					
Top 10% most highly disadvantaged	63 (4.3)	16 (3.8)	24 (4.1)	23 (5.1)	0.659
Highly disadvantaged	174 (11.9)	51 (12.1)	66 (11.4)	57 (12.6)	-
Moderate-highly disadvantaged	237 (16.1)	67 (15.9)	94 (16.2)	76 (16.7)	-
Low-moderately disadvantaged	222 (15.1)	73 (17.3)	77 (13.3)	72 (15.9)	-
Low disadvantaged	309 (21.0)	84 (19.9)	124 (21.4)	101 (22.2)	-
Top 10% least disadvantaged	451 (30.7)	131 (31.0)	195 (33.6)	125 (27.5)	-
Dietary intakes					
Energy, kJ/day	7097.4 ± 2077.6	6232.8 ± 1811.3	6945.4 ± 1924.6	8087.4 ± 2089.2	<0.001
Protein, g/day	79.5 ± 26.6	67.3 ± 22.0	78.1 ± 25.4	92.5 ± 26.1	<0.001
Calcium, mg/day	952.9 ± 345.4	869.7 ± 327.6	950.5 ± 342.7	1032.8 ± 347.2	<0.001
Alcohol, g/day	1.8 (0.3–9.8)	1.8 (0.3–9.3)	1.7 (0.3–9.3)	2.0 (0.3–10.4)	0.905

^1^ Data presented as mean ± SD, median (interquartile range) or number (*n*) and (%); ^2^ Vegetable serves were calculated based on the 2013 Australian Dietary Guidelines of a vegetable serve equal to 75 g/day; ^3^ *p* values are a comparison between groups using ANOVA, Kruskal-Wallis test, and Chi-square test where appropriate; ^4^ *n* = 1466; ^5^ *n* = 1467; ^6^ *n* = 1460; ^7^ *n* = 1456.

**Table 2:** Baseline characteristics according to fruit intake categories ^1^.

	Fruit Serve Intake ^2^			
	<1 Serves/Day	1 to <2 Serves/Day	≥2 Serves/Day	*p* Value ^3^
Number	417	560	491	
Age, years	74.9 ± 2.7	75.2 ± 2.7	75.3 ± 2.7	0.140
BMI ^4^, kg/m^2^	26.9 ± 4.6	27.3 ± 4.7	27.4 ± 4.9	0.257
Treatment (calcium) ^5^	210 (50.4)	279 (49.9)	266 (54.2)	0.335
Prevalent fracture ^5^	109 (26.2)	154 (27.5)	134 (27.3)	0.894
Prevalent diabetes mellitus	19 (4.6)	32 (5.7)	39 (7.9)	0.092
Physical activity ^4^, kJ/day	399.2 (0.0–806.6)	451.7 (70.6–811.6)	532.7 (210.2–928.8)	0.002
Smoked ever ^6^	178 (42.9)	175 (31.5)	193 (39.5)	0.001
Socioeconomic status ^7^				
Top 10% most highly disadvantaged	18 (4.4)	24 (4.3)	21 (4.3)	0.998
Highly disadvantaged	54 (13.1)	65 (11.7)	55 (11.3)	-
Moderate-highly disadvantaged	70 (16.9)	90 (16.2)	77 (15.8)	-
Low-moderately disadvantaged	63 (15.3)	82 (14.7)	77 (15.8)	-
Low disadvantaged	85 (20.6)	123 (22.1)	101 (20.7)	-
Top 10% least disadvantaged	123 (29.8)	172 (30.9)	156 (32.0)	-
Dietary intakes				
Energy, kJ/day	6829.9 ± 1948.7	6812.5 ± 2003.7	7649.7 ± 2158.5	<0.001
Protein, g/day	75.5 ± 24.9	76.2 ± 24.7	86.6 ± 28.5	<0.001
Calcium, mg/day	854.9 ± 306.2	952.1 ± 338.8	1037.2 ± 362.5	<0.001
Alcohol, g/day	2.6 (0.3–11.9)	1.8 (0.4–9.3)	1.2 (0.1–7.9)	0.008

^1^ Data presented as mean ± SD, median [interquartile range] or number (*n*) and (%); ^2^ Fruit serves were calculated based on the 2013 Australian Dietary Guidelines of a fruit serve equal to 150 g/day; ^3^ *p* values are a comparison between groups using ANOVA, Kruskal-Wallis test, and Chi-square test where appropriate; ^4^ *n* = 1466; ^5^ *n* = 1467; ^6^ *n* = 1460; ^7^ *n* = 1456.

**Table 3:** Hazard ratios for fracture-related hospitalisation by vegetable serve intake ^1^.

		All Participants	*p* Value	Vegetable Serve Intake			
				<2 Serves/Day	2 to <3 Serves/Day	≥3 Serves/Day	*p* for Trend ^2^
All fractures	Number	1468		424	584	460	
	Events, *n* (%)	415 (28.3)		133 (31.4)	172 (29.5)	110 (23.9)	
	Age-adjusted	0.85 (0.77, 0.94)	0.002	1.00 (Referent)	0.87 (0.69, 1.09)	0.67 (0.52, 0.87)	0.002
	Multivariable-adjusted ^3^	0.88 (0.79, 0.98)	0.024	1.00 (Referent)	0.88 (0.70, 1.11)	0.73 (0.55, 0.96)	0.023
Hip fractures	Events, *n* (%)	158 (10.8)		57 (13.4)	66 (11.3)	35 (7.6)	
	Age-adjusted	0.77 (0.65, 0.90)	0.002	1.00 (Referent)	0.78 (0.55, 1.12)	0.52 (0.34, 0.79)	0.002
	Multivariable-adjusted	0.82 (0.69, 0.98)	0.033	1.00 (Referent)	0.88 (0.61, 1.27)	0.61 (0.39, 0.97)	0.037

^1^ Hazard ratios (95% CI) for fracture-related hospitalisation by vegetable serve intake analysed using Cox proportional hazard models. Vegetable serves were calculated based on the 2013 Australian Dietary Guidelines of a vegetable serve equal to 75 g/day; ^2^ Test for trend conducted using median value for each vegetable serve category (1.6, 2.5, and 3.6 serves/day); ^3^ Multivariable-adjusted model included age, BMI, treatment code, prevalent diabetes mellitus, socioeconomic status, physical activity, smoking history, and energy, protein, calcium, and alcohol intake.

**Table 4:** Multivariable-adjusted hazard ratios for fracture-related hospitalisation by vegetable type ^1^.

	All Participants ^2^	*p* Value	Quartiles of Vegetable Types ^3^				
			Q1	Q2	Q3	Q4	*p* For Trend ^4^
All fractures							
Cruciferous	0.90 (0.81, 0.99)	0.026	1.00 (Referent)	0.79 (0.61, 1.04)	0.80 (0.61, 1.05)	0.72 (0.54, 0.95)	0.030
Allium	0.81 (0.68, 0.96)	0.013	1.00 (Referent)	0.89 (0.68, 1.16)	0.77 (0.59, 1.01)	0.66 (0.49, 0.88)	0.003
Yellow/orange/red	0.95 (0.88, 1.03)	0.184	1.00 (Referent)	0.83 (0.64, 1.09)	0.71 (0.54, 0.94)	0.80 (0.61, 1.06)	0.118
Leafy green	0.97 (0.82, 1.15)	0.772	1.00 (Referent)	0.80 (0.61, 1.05)	0.73 (0.55, 0.96)	0.84 (0.64, 1.10)	0.229
Legumes	0.94 (0.84, 1.05)	0.264	1.00 (Referent)	0.91 (0.70, 1.20)	0.82 (0.62, 1.08)	0.87 (0.66, 1.16)	0.332
Hip fractures							
Cruciferous	0.89 (0.76, 1.05)	0.157	1.00 (Referent)	0.80 (0.53, 1.22)	0.79 (0.51, 1.22)	0.65 (0.41, 1.04)	0.083
Allium	0.75 (0.56, 1.00)	0.050	1.00 (Referent)	0.74 (0.48, 1.14)	0.85 (0.56, 1.31)	0.61 (0.38, 0.99)	0.086
Yellow/orange/red	0.90 (0.79, 1.03)	0.130	1.00 (Referent)	1.05 (0.69, 1.61)	0.89 (0.57, 1.40)	0.75 (0.47, 1.22)	0.181
Leafy green	0.91 (0.69, 1.19)	0.484	1.00 (Referent)	1.09 (0.71, 1.67)	0.75 (0.47, 1.20)	0.94 (0.60, 1.47)	0.531
Legumes	0.87 (0.72, 1.05)	0.152	1.00 (Referent)	1.15 (0.75, 1.77)	0.88 (0.56, 1.37)	0.79 (0.49, 1.27)	0.188

^1^ Multivariable-adjusted hazard ratios (95% CI) for fracture-related hospitalisation by vegetable type analysed using Cox proportional hazard models, adjusted for age, BMI, treatment code, prevalent diabetes mellitus, socioeconomic status, physical activity, smoking history, and energy, protein, calcium, and alcohol intake; ^2^ Results are presented per 10 g/day for allium vegetables and per 20 g/day for all other types of vegetables; ^3^ Quartiles for cruciferous vegetables were Q1 (<15 g/day), Q2 (15–28 g/day), Q3 (29–44 g/day), Q4 (>44 g/day); allium vegetables were Q1 (<3 g/day), Q2 (3–6 g/day), Q3 (7–11 g/day), Q4 (>11 g/day); yellow/orange/red vegetables were Q1 (<32 g/day), Q2 (32–47 g/day), Q3 (48–68 g/day), Q4 (>68 g/day); leafy green vegetables were Q1 (<9 g/day), Q2 (9–16 g/day), Q3 (17–25 g/day), Q4 (>25 g/day); and legumes were Q1 (<15 g/day), Q2 (15–23 g/day), Q3 (24–36 g/day), Q4 (>36 g/day); ^4^ Test for trend conducted using median values of each quartile of vegetable type.

**Table 5:** Hazard ratios for fracture-related hospitalisation by fruit serve intake ^1^.

		All Participants	*p* Value	Fruit Serve Intake			
				<1 Serves/Day	1 to <2 Serves/Day	≥2 Serves/Day	*p* for Trend ^2^
All fractures	Number	1468		417	560	491	
	Events, *n* (%)	415 (28.3)		119 (28.5)	159 (28.4)	137 (27.9)	
	Age-adjusted	0.94 (0.84, 1.06)	0.333	1.00 (Referent)	0.93 (0.73, 1.18)	0.90 (0.70, 1.15)	0.412
	Multivariable-adjusted ^3^	0.99 (0.88, 1.12)	0.855	1.00 (Referent)	0.94 (0.74, 1.21)	0.97 (0.75, 1.25)	0.825
Hip fractures	Events, *n* (%)	158 (10.8)		50 (12.0)	60 (10.7)	48 (9.8)	
	Age-adjusted	0.86 (0.71, 1.03)	0.109	1.00 (Referent)	0.82 (0.46, 1.19)	0.73 (0.49, 1.09)	0.129
	Multivariable-adjusted	0.89 (0.73, 1.08)	0.242	1.00 (Referent)	0.81 (0.55, 1.19)	0.76 (0.50, 1.15)	0.207

^1^ Hazard ratios (95% CI) for fracture-related hospitalisation by fruit serve intake analysed using Cox proportional hazard models. Fruit serves were calculated based on the 2013 Australian Dietary Guidelines of a fruit serve equal to 150 g/day; ^2^ Test for trend conducted using median value for each fruit serve category (0.7, 1.5 and 2.5 serves/day); ^3^ Multivariable-adjusted model included age, BMI, treatment code, prevalent diabetes mellitus, socioeconomic status, physical activity, smoking history, and energy, protein, calcium, and alcohol intake.

**Table 6:** Multivariable-adjusted hazard ratios for fracture-related hospitalisation by fruit type.

	All Participants	*p* Value
All fractures		
Apples and pears	0.99 (0.96, 1.02)	0.698
Oranges and other citrus fruits	1.01 (0.97, 1.04)	0.710
Bananas	1.02 (0.97, 1.07)	0.386
Other fruits	0.98 (0.95, 1.02)	0.378
Hip fractures		
Apples and pears	0.99 (0.94, 1.04)	0.604
Oranges and other citrus fruits	0.99 (0.94, 1.05)	0.828
Bananas	0.97 (0.89, 1.05)	0.432
Other fruits	0.97 (0.91, 1.03)	0.279

Multivariable-adjusted hazard ratios (95% CI) for fracture-related hospitalisation by fruit type (per 20 g/day) analysed using Cox proportional hazard models, adjusted for age, BMI, treatment code, prevalent diabetes mellitus, socioeconomic status, physical activity, smoking history, and energy, protein, calcium, and alcohol intake.

## Supplementary Materials

The following is available online at www.mdpi.com/2072-6643/9/5/511/s1: Table S1: Descriptive statistics for intakes of cruciferous, allium, and total vegetables at baseline, 5 years (2003), and 7 years (2005), Table S2: Multivariable-adjusted hazard ratios for fracture-related hospitalisation for mean intakes of cruciferous, allium, and total vegetables across baseline, 5 years, and 7 years.
