# Supplementary material for: Vegetable and Fruit Intake and Fracture-Related Hospitalisations: A Prospective Study of Older Women
Source: Nutrients. 2017 May 18;9(5):511. doi: 10.3390/nu9050511 (PMC5452241; doi:10.3390/nu9050511)
Supplement: Supplementary file 1 [file nutrients-09-00511-s001.zip › nutrients-193388-Supplementary-Final.pdf]

nutrients

MDPI

Table S1. Descriptive statistics for intakes of cruciferous, allium, and total vegetables at baseline, 5 years (2003), and 7 years (2005).

	Mean	SD
Cruciferous vegetables		
Baseline	32.1	21.8
5 years (2003)	32.2	23.0
7 years (2005)	29.6	21.3
Allium vegetables		
Baseline	8.1	6.9
5 years (2003)	6.6	5.5
7 years (2005)	5.9	5.4
Total vegetables		
Baseline	200.7	77.4
5 years (2003)	178.4	74.3
7 years (2005)	169.2	68.7

Data presented as mean and SD, $n = 986$.

Table S2. Multivariable-adjusted hazard ratios for fracture-related hospitalisation for mean intakes of cruciferous, allium, and total vegetables across baseline, 5 years, and 7 years.

	All Participant ($n = 986$)	p-Value
Cruciferous vegetables	0.89 (0.77, 1.02)	0.095
Allium vegetables	0.83 (0.63, 1.10)	0.193
Total vegetables	0.84 (0.71, 0.99)	0.044

Multivariable-adjusted hazard ratios (95% CI) for fracture-related hospitalisation analysed using Cox proportional hazard models, adjusted for age, body mass index (BMI), treatment code, prevalent diabetes mellitus, socioeconomic status, physical activity, smoking history, and energy, protein, calcium, and alcohol intake. Results are presented per 20 g/day for cruciferous vegetables, per 10 g/day for allium vegetables, and per 75 g/day for total vegetables.
